# Functional analysis of three *BrMYB28* transcription factors controlling the biosynthesis of glucosinolates in *Brassica rapa*

**DOI:** 10.1007/s11103-016-0437-z

**Published:** 2016-01-28

**Authors:** Mi-Suk Seo, Mina Jin, Jin-Hyuk Chun, Sun-Ju Kim, Beom-Seok Park, Seong-Han Shon, Jung Sun Kim

**Affiliations:** 1 Genomics Division, Department of Agricultural Bio-resources, National Academy of Agricultural Science, Rural Development Administration (RDA), Wansan-gu, Jeonju, Korea; 2 Department of Biological Environment and Chemistry, College of Agriculture and Life Science, Chungnam National University, Yuseong-gu, Daejeon, Korea

**Keywords:** *Brassica rapa*, Glucosinolates, Transcription factor *BrMYB28*s, Chinese cabbage, Transgenic plats

## Abstract

Glucosinolates (GSLs) are secondary metabolites that have anticarcinogenic activity and play defense roles in plants of the Brassicaceae family. MYB28 is known as a transcription factor that regulates aliphatic GSL biosynthesis in *Arabidopsis thaliana*. Brassicaceae plants have three orthologous copies of *AtMYB28* derived from recent genome triplication. These *BrMYB28* genes have a high level of sequence homology, with 81–87 % similarities in the coding DNA sequence compared to *Arabidopsis*. Overexpression of three paralogous *BrMYB28* genes in transgenic Chinese cabbage increased the total GSL content in all T_1_ generation plants and in two inbred lines of homozygous T_2_ plants. The highest total GSL contents were detected in homozygous T_2_ lines overexpressing *BrMYB28.1,* which showed an approximate fivefold increase compared to that of nontransgenic plants. The homozygous T_2_ lines with overexpressed *BrMYB28.1* also showed an increased content of aliphatic, indolic, and aromatic GSLs compared to that of nontransgenic plants. Furthermore, all of the three *BrMYB28* genes were identified as negative regulators of *BrAOP2* and positive regulators of *BrGSL*-*OH* in the homozygous T_2_ lines. These data indicate the regulatory mechanism of GSL biosynthesis in *B. rapa* is unlike that in *A. thaliana*. Our results will provide useful information for elucidating the regulatory mechanism of GSL biosynthesis in polyploid plants.

**Electronic supplementary material:**

The online version of this article (doi:10.1007/s11103-016-0437-z) contains supplementary material, which is available to authorized users.

## Introduction

Plants produce various secondary metabolites that play roles in plant defense against environmental change or stress, but are unrelated to the primary functions of plants, such as development, reproduction, and photosynthesis. More than 200,000 plant secondary metabolites are known, which perform useful, although not necessarily essential, functions in plant survival (Verpoorte et al. 2002; Yonekura-Sakakibara and Saito 2009). One of the largest known groups of secondary metabolites in the Brassicaceae family is the glucosinolates (GSLs)—compounds derived from amino acids and sugars (Hayes et al. 2008). GSLs are classified into three groups: aliphatic, derived from Met, Ala, Leu, Ile, or Val; indolic, derived from Trp; and aromatic, derived from Phe or Tyr (Kliebenstein et al. 2001). The biosynthesis of the three types of GSLs generally occurs via three stages. The precursor amino acids are chain-elongated in the early stage, and then converted into the core GSL structure during the second stage. The final stage involves side chain modification of the GSL structure (Zang et al. 2009). The compounds produced by the GSL biosynthesis pathway have recently attracted a high level of research interest, owing to their various beneficial activities, such as anticarcinogenic and antioxidative activity in mammals, and defense against pests and pathogens in plants (Sawada et al. 2009; Yonekura-Sakakibara and Saito 2009). Therefore, an understanding of the regulation of GSL biosynthesis will provide useful information for the study of plant secondary metabolism, and thereby improvements in the value of agricultural crops.

Studies of the mechanisms underlying GSL biosynthesis have mostly been performed in the model plant *Arabidopsis thaliana* using a molecular biology approach (Kliebenstein et al. 2001; Skirycz et al. 2006; Sønderby et al. 2010). Recently, genes controlling GSL biosynthesis have been identified in *Arabidopsis*. Certain R2R3 MYB transcription factors are known to participate in GSL regulation in *Arabidopsis* (Sønderby et al. 2010). For example, MYB28 has been identified as a positive regulator of aliphatic GSLs and high transcription levels of the MYB28 are associated with the production of large amounts of aliphatic GSLs (Hirai et al. 2007). Furthermore, the transcription factors MYB29, MYB51, and MYB76 have recently been shown to regulate the biosynthesis of indolic GSLs (Gigolashvili et al. 2007; Sønderby et al. 2007).

Recently, studies on GSL content regulation have investigated candidate genes using a genetic approach. The *RsMAM3*, *RsIPMDH1,* and *RsBCAT4* genes as candidate genes controlling 4-MTB-GSL content were identified in radish roots by QTL analysis using SNP markers developed by next-generation sequencing (Zou et al. 2013). The transcription factor MYB28 (*HAG1*), which participates in aliphatic GSL biosynthesis, was also identified by QTL analysis using associative transcriptomics by association between genetic variation and trait variation in the seeds of *Brassica napus* (Harper et al. 2012). However, although some studies have identified a few candidate genes and QTLs controlling GSL contents, functional validation of the candidate genes is very limited in Brassica crops owing to the difficulty of genetic modification.

The *B. rapa* genome has undergone genome triplication. A representative late flowering *BrFLC*s genes was obtained by selecting five *BrFLC* genes for sequence comparisons of triplication blocks for At5_3Mb (Kim et al. 2006; Yang et al. 2006). In addition, the genome sequence project for *B. rapa* as model for polyploid genome studies was undertaken (Wang et al. 2011). The *B. rapa* genome sequence provides an important resource for studying the evolution of polyploid genomes and a foundation for the genetic improvement of *Brassica* oil and vegetable crops.

Using the *B. rapa* genome sequence, many genes related to GSL biosynthesis pathways were identified by comparative genome analysis with *Arabidopsis* (Zang et al. 2009). The results predictably suggested that the complexity of GSL biosynthesis regulation in *B. rapa* is greater than that in *Arabidopsis* because of the polyploid *B. rapa* genome. Polyploidy of genomes is a very important event for plant evolution and diversity. Polyploidy leads to amplification or redundancy of genes, and duplicated genes with different expression patterns, such as silencing or strong expression, have arisen compared with the ancestral function. Allopolyploid plants also show various plant-specific expression patterns of genes duplicated by polyploidy (Udall et al. 2006).

The existence of multiple paralog genes in the polyploid *B. rapa* genome suggests the possibility for variation of expression and function among the paralogs of candidate genes. Although functional studies for candidate GSL biosynthesis genes have been performed in a few Brassica crops, the functional analysis of paralog genes in *B. rapa* is currently still very limited (Augustine et al. 2013). Moreover, no transgenic *B. rapa* has been produced for paralog genes related to GSL biosynthesis because of technical difficulties associated with the genetic transformation of *B. rapa*.

The MYB28 transcription factor, which is known to be a key regulator of aliphatic GSL biosynthesis, has been determined to have three paralogs, as a result of genome triplication in *B. rapa* (Zang et al. 2009). In the present study, we identified the three *BrMYB28* transcription factors (designated *BrMYB28.1, BrMYB28.2,* and *BrMYB28.3*) and characterized their gene structure and variation in expression. Furthermore, in a functional study, we successfully over-expressed the three paralogous *BrMYB28* transcription factors in transgenic *B. rapa*. Understanding the structure and function of the *BrMYB28* transcription factors will facilitate analysis of the mechanisms underlying GSL biosynthesis in *B. rapa* using a molecular approach.

## Materials and methods

### Isolation of three *BrMYB28* genes from *B. rapa*

Genomic information on the GSL biosynthesis-related transcription factors of *B. rapa* was obtained from the National Center for Biotechnology Information (NCBI) and the *Brassica* Database (http://brassicadb.org/brad/). PCR amplification was performed on total genomic DNA of the ‘Chiifu’ cultivar of *B. rapa* using gene-specific primers designed based on genomic information. The gene-specific primers of the three *BrMYB28* genes were designed for gateway cloning (Invitrogen, USA). PCR was performed with 30 cycles (94 °C for 30 s, 52 °C for 30 s, and 72 °C for 1.5 min) and a final extension at 72 °C for 10 min. PCR products were cloned into a pDONR221 vector to prepare an entry clone through the use of BP clonase (Invitrogen, USA). After transformation into *Escherichia coli* DH5α competent cells, plasmids were isolated and genomic sequences were validated using an ABI 3730xl sequencer. The gene structures were predicted by sequence comparison with the *Arabidopsis* sequence. The *BrMYB28* genes with complete coding sequence were analyzed for molecular characterization, and the R2R3 binding domain was predicted by SMART analysis (http://smart.embl-heidelberg.de/). A multiple alignment analysis of *BrMYB28* and *AtMYB28* was performed using ClustalW2. A phylogenetic tree was obtained using MEGA6 software based on the previously published MYB transcription factors related to the GSL biosynthesis pathway in *B. rapa* and *A. thaliana*.

### Expression analysis of *BrMYB28* transcription factors

Total RNA was isolated from various organs of *B. rapa* ‘Chiifu’ using an RNeasy mini kit (Qiagen, USA), and treated with RNase-free DNase I (Takara, Japan) to eliminate contaminated genomic DNA. One microgram of total RNA was used as a template for RT-PCR using an AMV one-step RT-PCR kit (Takara, Japan). The primers for member-specific detection of the expression of *BrMYB28s* were respectively designed for the 5′ and 3′ UTR regions. The *BrActin* gene primer was used as a control for all expression analyses. All the specific primers used in this study are listed in supplementary Table S1. The PCR reaction comprised predenaturating at 94 °C for 5 min, followed by 35 cycles of denaturation (94 °C for 30 s), annealing (52 °C for 30 s), and extension (72 °C for 1.5 min), and with a final extension of 10 min at 72 °C. The PCR products were visualized on a 1.2 % agarose gel stained with ethidium bromide. The RT-PCRs were performed with two replicates as a check for experimental fidelity. The expression profile of *BrMYB28* genes related to the developmental stages in *B. rapa* was examined by analyzing the microarray and unigene databases (http://nabic.rda.go.kr).

### Genetic transformation of Chinese cabbage

Two inbred lines (NW and CT001) of Chinese cabbage (*B. rapa* ssp. *pekinensis*), i.e., plant materials for which successful transformation was previously reported (Min et al. 2007; Park et al. 2011), were used. These lines were kindly provided by the Nong Woo Bio Co. (NW) and Carrotop Seed Company (CT001) in Korea. Mature seeds were surface-sterilized in 70 % ethanol for 1 min and in 2 % sodium hypochlorite for 20 min, and then rinsed 3 times with sterile distilled water. Seeds were placed on 1/2 MS medium (Murashige and Skoog 1962) solidified with 7.5 g/L plant agar. The plates were incubated at 25 °C in the dark for 5 days. The hypocotyls germinated from the mature seeds were cut to a length of approximately 8 mm and placed on MS medium supplemented with 3 mg/L BA (6-benzylaminopurine), 1 mg/L NAA (1-naphthalene acetic acid) and solidified with 7 g/L plant agar. Hypocotyls were incubated at 22 °C under a 16/8 h light/dark photoperiod for 3 days.

For transformation with the three *BrMYB28* genes, the clones within the pDONR221 entry vector (obtained as described above) were inserted into a pH2GW7 binary vector using LR clonase (Invitrogen, USA). The binary vector contains a selectable hygromycin resistance marker gene. The disarmed *Agrobacterium tumefaciens* strain GV3101 containing the *BrMYB28* constructs were used for transformation (Fig. 4A). *Agrobacterium* carrying BrMYB28 TFs were grown at 28 °C in the dark for 24 h on LB medium supplemented with 100 mg/L spectinomycin and harvested by centrifugation. The bacterial pellets were resuspended in MS suspension medium containing 36 g/L glucose and 5 mg/L acetosyringone, and then the hypocotyl explants were immersed in the bacterial suspension for 20 min. The explants were co-cultivated on co-culture medium (MS medium containing 3 mg/L BA, 1 mg/L NAA, 1 mg/L AgNO_3_, and 5 mg/L acetosyringone) for 3 days at 23 °C in the dark. After co-cultivation, the explants were rinsed three times in distilled water containing 250 mg/L cefotaxime to kill the *Agrobacterium*. The explants were transferred to selection medium (MS medium containing 3 mg/L BA, 1 mg/L NAA, and 1 mg/L AgNO_3_, 250 mg/L cefotaxime, and 10 mg/L hygromycin) and then incubated at 23 °C under a 16/8 h light/dark photoperiod. Hygromycin-resistant shoots that regenerated from the hypocotyls were transferred to an MS regeneration medium (MS medium containing 3 mg/L BA, 0.1 mg/L NAA, 1 mg/L AgNO_3_, 250 mg/L cefotaxime, and 10 mg/L hygromycin) for shoot elongation. The regenerated hygromycin-resistant plantlets were transferred to MS hormone-free medium containing 150 mg/L cefotaxime, and 10 mg/L hygromycin for further root development. The regeneration was carried out at 23 °C under a 16/8 h light/dark photoperiod. The hygromycin-resistant plantlets were transferred to soil in pots and grown to maturity in a greenhouse. Genomic DNA was extracted from the leaves of nontransformant and hygromycin-resistant plants. Hygromycin-resistant T_0_ plants were identified from the insertion of *hpt* and *BrMYB28* genes by PCR using gene-specific primers (Supplementary Table 1). PCR for the three *BrMYB28* genes was performed using the gene-specific forward and reverse primers of the 35S terminator. The PCR was performed in a thermal cycler using the following amplification conditions: 35 cycles of 30 s at 94 °C, 30 s at 52 °C, and 2 min at 72 °C. The primers used for the 757-bp *hpt* gene fragment were 5′-ATTCCGGAAGTGCTTGACAT-3′(forward) and 5′-CGGCGAGTACTTCTACACAGC-3′(reverse). Thermal cycling conditions for the *hpt* gene were as follows: 30 cycles of 30 s at 94 °C, 30 s at 58 °C, and 1 min at 72 °C.

### Production of T_1_ and T_2_ progeny

T_0_ plants were vernalized at 4 °C and the T_1_ seeds were obtained by self-crossing. The T_1_ seeds were surface-sterilized and then grown on MS medium supplemented with hygromycin (20 mg/L). Hygromycin-resistant T_1_ transgenic seedlings were transplanted into soil. The T_1_ progenies with a segregation ratio of 3 (resistant):1(sensitive) determined by the χ^2^ test were self-crossed and T_2_ seeds were obtained. The T_2_ seeds were germinated on MS medium containing hygromycin (20 mg/L) for the selection of homozygous transgenic lines, and homozygous T_2_ transgenic hygromycin-resistant seedlings geminated from all of the seeds. All of the T_1_ and T_2_ plants were identified to contain the inserted *hpt* gene and *BrMYB28* genes by PCR analysis.

### Gene expression in transgenic plants

Real-time PCR was carried out to investigate the transcription levels of *BrMYB28* genes and the GSL structural genes in transgenic Chinese cabbage. Total RNA was isolated as described above. Approximately 2 µg of total RNA was reverse transcribed into cDNA with oligo-dT primers using a first-strand cDNA synthesis kit (Genedepot). The synthesized cDNAs were diluted 10 times in sterilized water and real-time PCR was performed using 2 µL of diluted cDNA in 20 µL using SYBR Green mix (Geneall). The primers for member-specific detection of the expression of *BrMYB28s* were designed for the 3′ terminal region. The gene-specific primers used for PCR analysis are shown in Supplementary Table S1. We used the thermal cycler conditions recommended by the manufacturer as follows: 95 °C for 10 min, 55 °C for 30 s, and 72 °C for 30 s, with a final extension at 72 °C for 3 min.

### HPLC analysis of GSL contents

Desulfo (DS)-GSLs were extracted according to the procedure of Kim et al. (2007) and ISO 9167-1 (1992). Fresh leaves of 50-day-old plants were ground with a mortar and pestle into fine powder, and freeze-dried materials (100 mg) were extracted twice in 70 % methanol. As an external standard, we used 0.5 mg of sinigrin dissolved in 5 mL ultrapure water. The crude extracts were loaded on Sephadex A25 columns and desulfated overnight using aryl sulfatase (E.C.3.1.6.1) prior to HPLC. DS-GSLs were analyzed using a 1200 series HPLC system (Agilent Technologies, CA, USA) equipped with an Inertsil ODS-3 column [150 × 3.0 mm ID, particle size 3 µm (GL Science, Tokyo, Japan)]. The HPLC analysis was carried out using a flow rate of 0.4 mL/min at a column oven temperature of 35 °C and a wavelength of 227 nm. The individual GSLs were quantified by comparison with the external standard sinigrin, and the values for total GSLs were obtained by summing the values of the individual GSLs identified (Supplementary Table S3).

## Results

### Genomic sequence analysis of three *MYB28s* isolated from *B. rapa*

The three orthologous copies in *B. rapa* corresponding to *AtMYB28* identified by comparative analysis with *Arabidopsis* using the *Brassica* Database (http://brassicadb.org/brad/) in Table 1. The sequences of the three genes were as follows: 1350 nucleotides long for *BrMYB28.1*, 1378 for *BrMYB28.2*, and 1618 for *BrMYB28.3.* These are all longer than the sequence of *AtMYB28*. The three *BrMYB28* transcription factors of *B. rapa* showed 81–87 % sequence homology with *AtMYB28* (Supplementary Fig. 1). The structures of three *BrMYB28* genes comprise 3 exons and 2 introns, which is comparable to *AtMYB28*. The first and second exons have a very similar size (133 and 130 nucleotides, respectively) and show orthologous and paralogous sequence variation. These three paralogous genes anchored BAC clones with KBrB034G03, KBrB051M06, and KBrH005L20, respectively, and are located on the A03, A09, and A02 chromosomes of *B. rapa*. After divergence of *Arabidopsis* and Brassicaceae and triplication of the Brassica genome, these genes may have undergone sequence substitutions, as well as insertion and deletion.

**Table 1:** Comparison of the *BrMYB28* TFs related glucosinolate biosynthesis with the Arabidopsis orthologs

Gene name	Length (bp)		No. of exsons [length (bp)]	No. of introns [length (bp)]	Chromosome position of *B. rapa*	Corresponding *B. rapa*	
	Gene	CDS				BAC clone	EST clone
*AtMYB28*(AT5G61420^a^)	1321	1101	3 (133,130,838)	2 (80,140)	–	–	–
*BrMYB28.1*(Bra012961)	1350	1065	3 (133,130,802)	2 (89,196)	A03	KBrB034G03	KBLS-095C01
*BrMYB28.2*(Bra035929)	1378	1074	3 (133,130,811)	2 (166,138)	A09	KBrB051M06	KBFL-120H07
*BrMYB28.3*(Bra029311)	1618	1119	3 (133,130,856)	2 (83,416)	A02	KBrH005L20	KFFB-103G11

^a^Arabidopsis genome ID of NCBI and *Brassica rapa* gene ID of BRAD

### Amino acid sequence comparison of *BrMYB28* genes

**Fig. 1:**
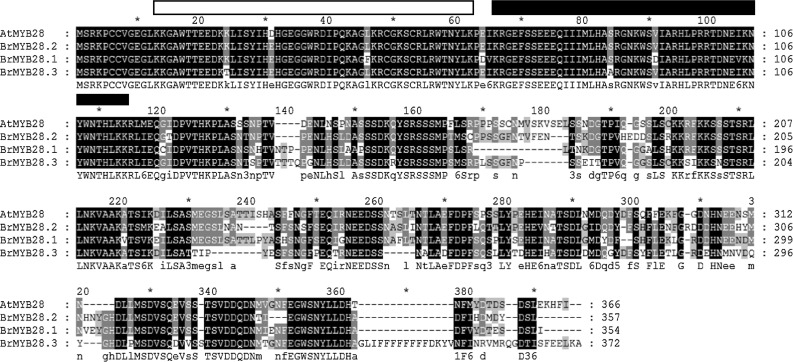
Amino acid sequence alignment of MYB28 proteins that regulate the glucosinolate biosynthesis pathway of *B. rapa* and *A. thaliana*. The R2 and R3 binding domains are boxed in *white* and *black*, respectively

**Fig. 2:**
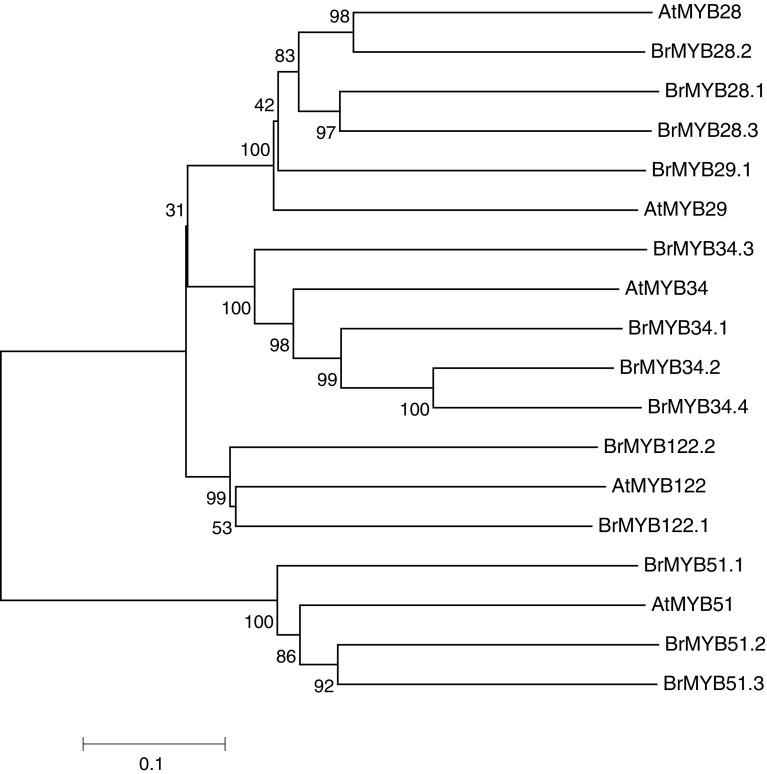
Phylogenetic analysis of MYB TFs related to the glucosinolate biosynthesis pathway in *B. rapa* and *Arabidopsis*. This tree was constructed using MEGA, version 6, software. Bootstrap values with 1000 replicates are denoted as percentages

Alignment and phylogenetic analyses of the three BrMYB28 proteins indicated that they are highly conserved. Multi-alignment revealed that they have two typical R2R3 MYB-DNA-binding domains (Fig. 1). An analysis of the deduced amino acid sequences indicated that *BrMYB28.1*, *BrMYB28.2,* and *BrMYB28.3* contain conserved R2R3 repeat MYB-DNA binding domains that share high amino acid sequence similarities of 94–99 % with *AtMYB28*. In contrast, the C-terminal region was shown to be highly polymorphic. These results indicate that amino acid sequence variations in the C-terminal region have led to the structural divergence of the *BrMYB28* transcription factors. The structural divergence of the three *BrMYB28* genes suggests the possibility of functional divergence of these genes in *B. rapa*. A phylogenetic tree was constructed using the deduced amino acid sequences of 14 MYB transcription factors related to the GSL biosynthesis pathways in *A. thaliana* and *B. rapa* (Fig. 2). In this tree, *BrMYB28.1* is more related to *BrMYB28.3* than to *BrMYB28.2*. *BrMYB28.1* and *BrMYB28.3* proteins were clustered in a small subgroup, whereas *AtMYB28* was clustered with *BrMYB28.2*. The subgroup with *AtMYB29* and *BrMYB29.1* forms a distinct large group. Therefore, the high homology of the *BrMYB28* and *BrMYB29* transcription factors in this large group indicates that they are evolutionary conserved and closely related.

### Differential expression of *BrMYB28* genes in *B. rapa*

**Fig. 3:**
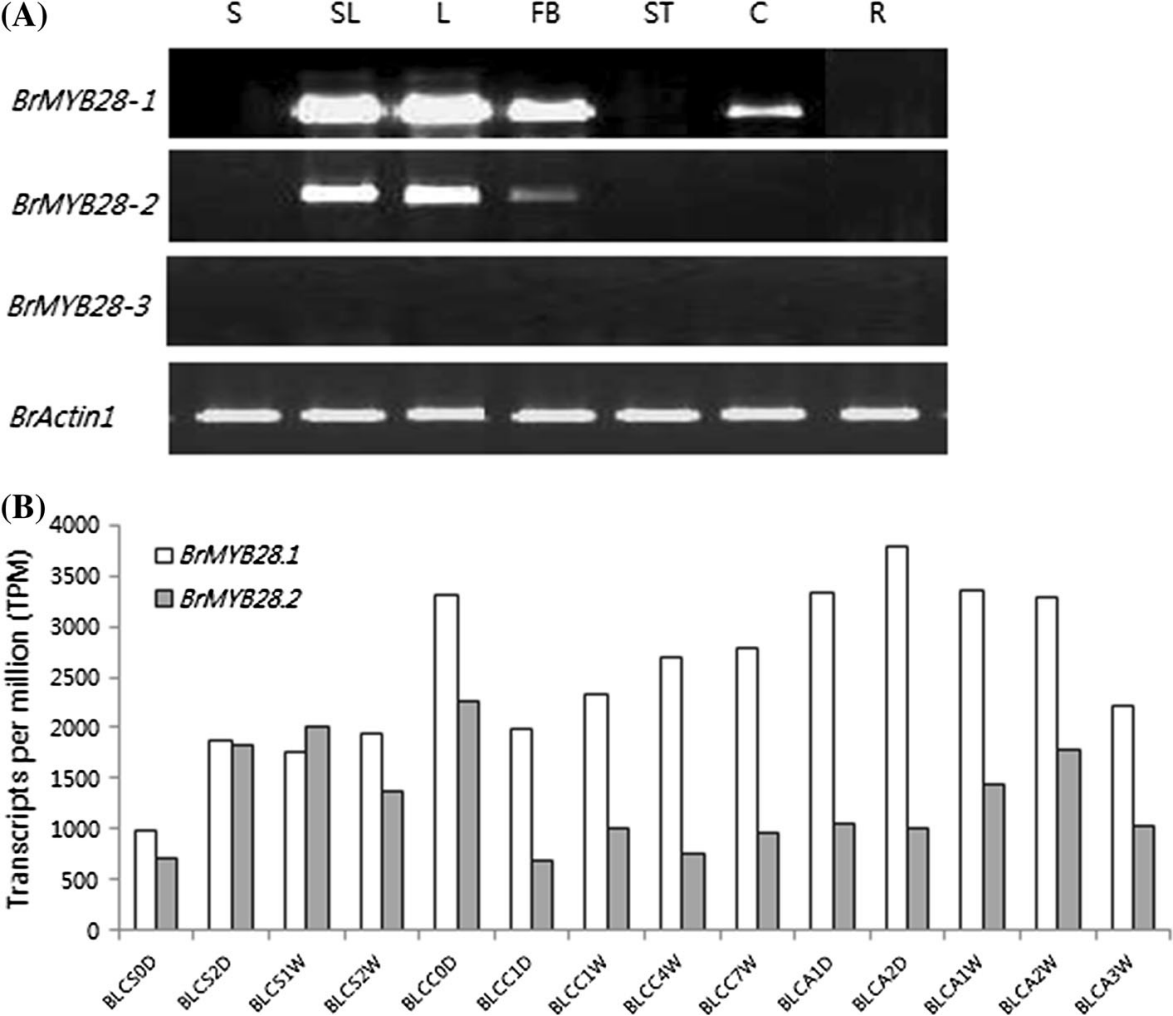
Expression analysis of *BrMYB28* TFs in various organs and developmental stages. **A** RT-PCR analysis of *BrMYB28* TFs in different types of tissues. S, seed; SL, seedling; L, mature leaf (3-week-old vegetative stage); FB, floral bud; ST, stamen; C, carpel; R, root. The PCR products are approximately 1 kb for *BrMYB28* genes. *BrActin1* is approximately 500 bp and serves as an internal control. **B** Microarray expression analysis of *BrMYB28* TFs in different growth stages. BLCS0D, seeds; BLCS2D, seedling (2 days old); BLCS1W, whole plant, 1-week-old vegetative stage (7 days old); BLCS2W, whole plant, 2-week-old vegetative stage (14 days old); BLCC0D, whole plant, 3-week-old vegetative stage (21 days old); BLCC1D, whole plant, 1 day after light chilling at 4 °C (22 days old); BLCC1W, whole plant, 1 week after light chilling at 4 °C (28 days old); BLCC4W, whole plant, 4 weeks after light chilling at 4 °C (56 days old); BLCC7W, whole plant, 7 weeks after light chilling at 4 °C (70 days old); BLCA1D, whole plant, 1 day after greenhouse growth (71 days old); BLCA2D, whole plant, 2 days after greenhouse growth (72 days old); BLCA1W, whole plant, 1 week after greenhouse growth (77 days old); BLCA2W, whole plant, 2 weeks after greenhouse growth (84 days old); BLCA3W, whole plant, 3 weeks after greenhouse growth (91 days old)

To identify the transcription levels of *BrMYB28* genes in various organs and developmental stages of *B. rapa*, the expression patterns of these genes were analyzed by RT-PCR and microarray (Fig. 3). The *BrMYB28.3* gene was not expressed in all organs and growth stages. The expression patterns of *BrMYB28.1* and *BrMYB28.2* were similar in all organs except the carpels (Fig. 3A). These two genes were most highly expressed in leaves, but showed no expression in seeds, stamens, and roots. The *BrMYB28.1* gene showed the highest transcription levels in various organs of *B. rapa*. The expression profiles of *BrMYB28.1* and *BrMYB28.2* in different developmental stages of *B. rapa* analyzed using the microarray database is shown in Fig. 3B. The expression of both these genes was observed in most developmental stages. Maximum expression was detected at 72 days for *BrMYB28.1* and 21 days for *BrMYB28.2*. *BrMYB28.1* was more highly expressed than *BrMYB28.2* in various developmental stages, consistent with the results of RT-PCR analysis. Expression of the two genes was markedly decreased by chilling treatment; however, the expression levels of *BrMYB28.1* increased with time. Higher levels of *BrMYB28.2* expression were detected in vegetative stages (BLCS2D–BLCC0D) than in reproductive stages (BLCA1D–BLCA3W), whereas for *BrMYB28.1,* higher expression levels were detected in the reproductive stages. Therefore, these paralogous *BrMYB28* genes have different expression patterns in various organs and developmental stages.

### Genetic transformation of the three *BrMYB28* genes in Chinese cabbage

**Fig. 4:**
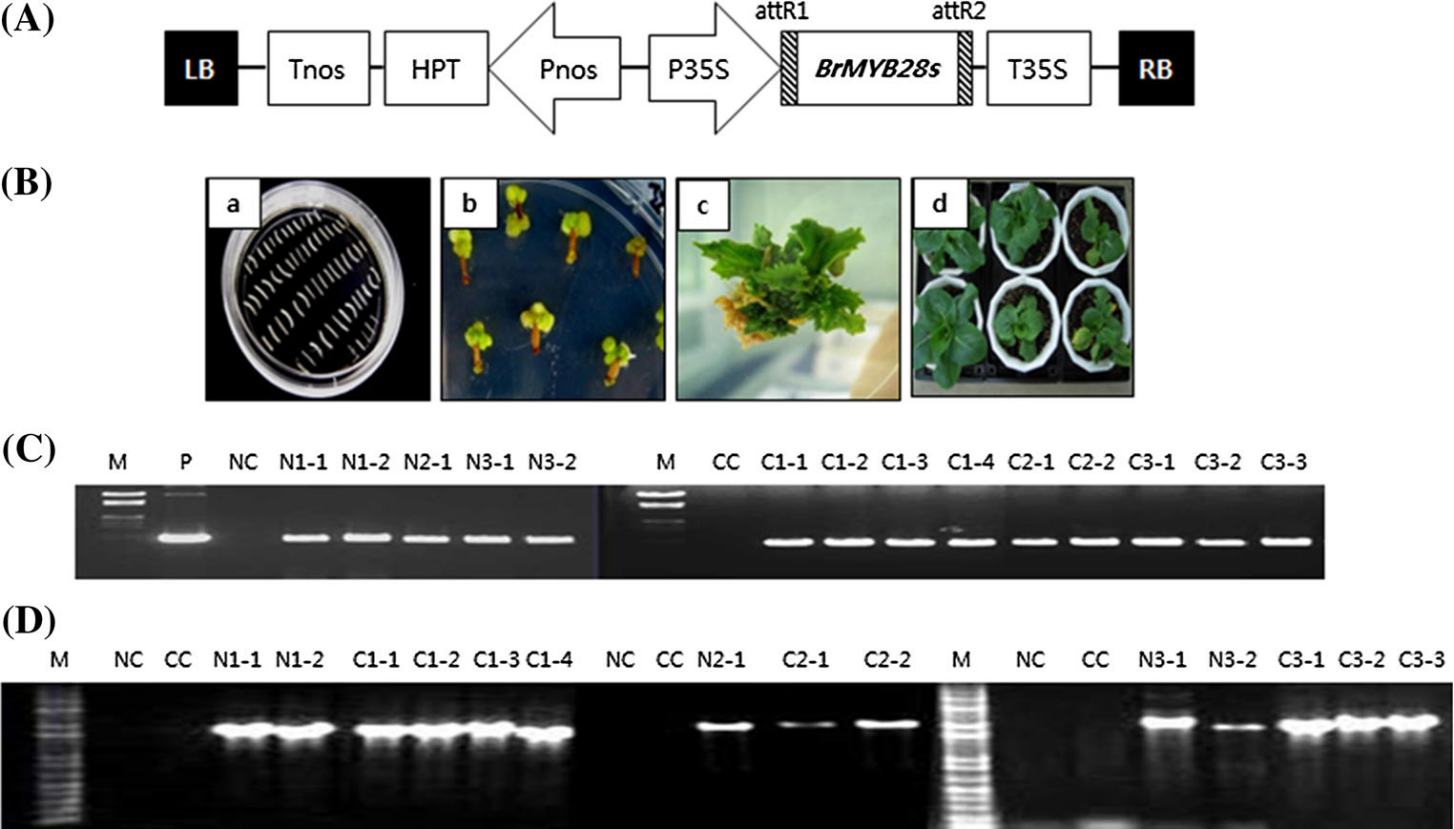
Schematic diagram of part of the T-DNA region of the binary vector and PCR analysis of transgenic Chinese cabbage. **A** T-DNA region of binary vector construct used for *Agrobacterium*-mediated transformation. LB, left border; RB, right border; 35S Pro, CaMV 35S promoter; Pnos, Nos promoter; Tnos, Nos terminator, HPT, hygromycin resistance gene. **B** *Agrobacterium*-mediated transformation of *BrMYB28* genes in Chinese cabbage. **a** Hypocotyl explants of *B. rapa* used for transformation. **b** Hygromycin-resistant callus induced from hypocotyl in selection medium containing 10 mg/L hygromycin. **c** Hygromycin resistance shoot regenerated from callus in regeneration medium containing 10 mg/L hygromycin. **d** The hygromycin-resistant plantlets were transferred to soil in pots and grown to maturity in a greenhouse with non-transgenic plants (leftmost panel). **C**, **D** Detection of the *hpt* gene (C) and the three *BrMYB28* genes (D) in hygromycin-resistant plants (T_1_) by PCR analysis. The PCR products were identified at 757 bp for the *hpt* gene and 1454 bp for *BrMYB28.1*, 1581 bp for *BrMYB28.2*, and 1821 bp for *BrMYB28.3*. M, molecular weight marker; P, plasmid DNA; NC, CC, nontransgenic NW line (NC) and CT001 line (CC); N1-1,2, NW *BrMYB28.1* gene transgenic plants; N2-1, NW *BrMYB28.2* gene transgenic plant; N3-1,2, NW *BrMYB28.3* gene transgenic plants; C1-1–4, CT001 *BrMYB28.1* gene transgenic plants; C2-1–2; CT001 *BrMYB28.2* gene transgenic plants; C3-1–3, CT001 *BrMYB28.3* gene transgenic plants

The three *BrMYB28* genes were transferred into two different Chinese cabbage inbred lines using *Agrobacterium*-mediated transformation (Fig. 4B). The hypocotyls were infected by co-cultivation with *Agrobacterium* containing each of the three *BrMYB28* genes, and shoots were regenerated in selection medium containing 10 mg/L hygromycin. The hygromycin-resistant plants were obtained after 3 months in regeneration medium and grown to maturity in a greenhouse. T_1_ seeds were obtained from the self-crossed T_0_ plants, and the segregation ratio was determined in MS medium containing 20 mg/L hygromycin (Supplementary Table 2). To identify gene integration into T_1_ plants with a segregation ratio of 3:1, PCR amplification was performed (Fig. 4C). PCR amplification of the 757-bp *hpt* gene products was observed in all hygromycin-resistant plants, whereas this band was not detected in nontransgenic plants. T_1_ plants containing *hpt* were also identified by PCR amplification of *BrMYB28* genes containing the 35S terminator (Fig. 4D). The T_1_ plants were self-crossed and T_2_ seeds were germinated on MS medium containing hygromycin for selection of transgenic homozygous lines (Supplementary Table S2). The T_2_ homozygous lines were obtained as nonsegregated resistant plants in all T_1_ plants with a segregation ratio of 3:1. T_1_ and T_2_ transgenic plants overexpressing *BrMYB28* genes showed normal phenotypes compared to the nontransgenic plants (Fig. 4B–d). However, the homozygous T_2_ plants obtained from C2-1 and C2-2 transgenic T_1_ plants did not grow well. In our future studies, we will further attempt to obtain homozygous T_2_ plants from C2-1 and C2-2.

### GSL content variation in T_1_ transgenic plants containing overexpressed *BrMYB28* genes

**Fig. 5:**
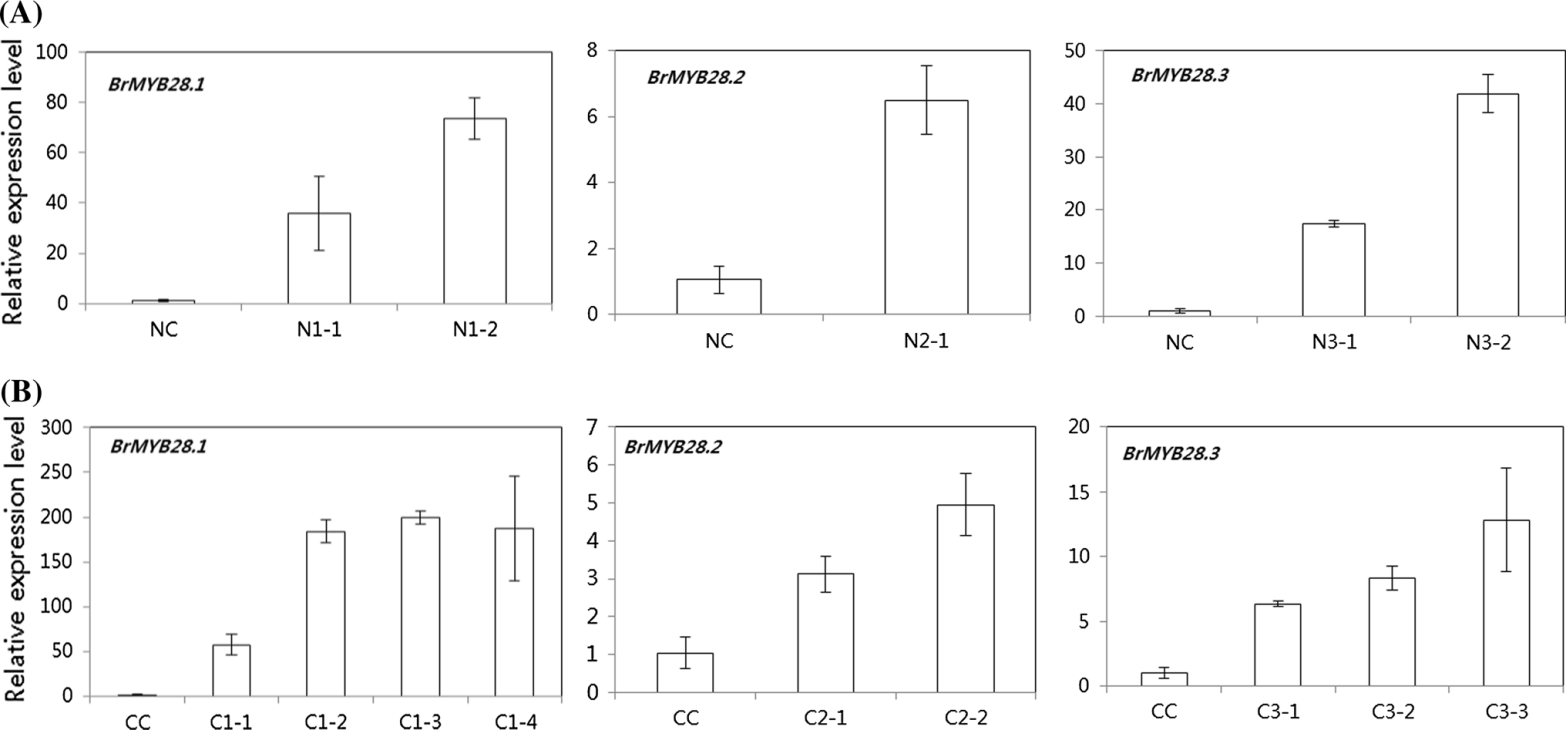
Expression of genes involved in glucosinolate biosynthesis in the 6-week-old leaves of T_1_ transgenic NW (**A**) and CT001 (**B**) plants. Relative expression was determined in triplicate measurements in three independent biological replicates. The *Bractin* gene was used as a quantitative control. NC, CC, non-transgenic plants; N1-1,2, NW *BrMYB28.1* gene transgenic plants; C1-1–4, CT001 *BrMYB28.1* gene transgenic plants; N2-1, NW *BrMYB28.2* gene transgenic plant; C2-1–3, CT001 *BrMYB28.2* gene transgenic plants; N3-1,2, NW *BrMYB28.3* gene transgenic plants; C3-1–3, CT001 *BrMYB28.3* gene transgenic plants

**Fig. 6:**
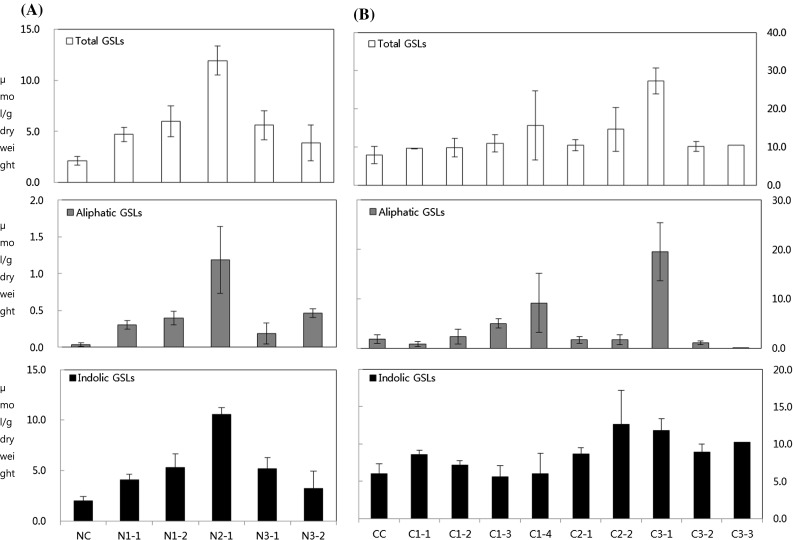
HPLC analysis of GSL content in leaves of T_1_ transgenic NW (**A**) and CT001 (**B**) plants. Values are the means of 3 replications. Bars represent the standard error of the mean. NC, CC, non-transgenic plants; N1-1, 2, NW *BrMYB28.1* gene transgenic plants; C1-1–5, CT001 *BrMYB28.1* gene transgenic plants; N2-1, NW *BrMYB28.2* gene transgenic plant; C2-1, 2, CT001 *BrMYB28.2* gene transgenic plants; N3-1,2, NW *BrMYB28.3* gene transgenic plants; C3-1–3, CT001 *BrMYB28.3* gene transgenic plants

Six-week-old leaves were used for analysis of transcript levels and GSL content in *BrMYB28* transgenic plants. Real-time PCR demonstrated that the *BrMYB28* genes were overexpressed efficiently in all of the T_1_ transgenic Chinese cabbage plants (Fig. 5). Significantly up-regulated expression of *BrMYB28* genes was observed in all transgenic plants compared to nontransgenic plants; moreover, the expression levels varied in the transgenic plants. The relative gene expression levels were increased by approximately 35- to 185-fold for *BrMYB28.1*, 5- to 8.5-fold for *BrMYB28.2,* and 8- to 41-fold for *BrMYB28.3* compared with the nontransgenic plants. To determine which GSLs are increased in transgenic plant, we measured GSL accumulation in T_1_ transgenic plants by HPLC analysis. The overexpression of *BrMYB28* genes in transgenic plants elevated the accumulation of total GSLs by 1.8- to 5.6-fold for NW lines and 1.2- to 2.4-fold for CT001 lines compared with the nontransgenic plants (Fig. 6, Supplementary Table S4). Both aliphatic and indolic GSLs were found to be elevated in NW transgenic plants. In contrast, the aliphatic GSLs were decreased in some CT001 transgenic plants.

### Expression analysis of genes related to GSL biosynthesis in homozygous T_2_ lines

**Fig. 7:**
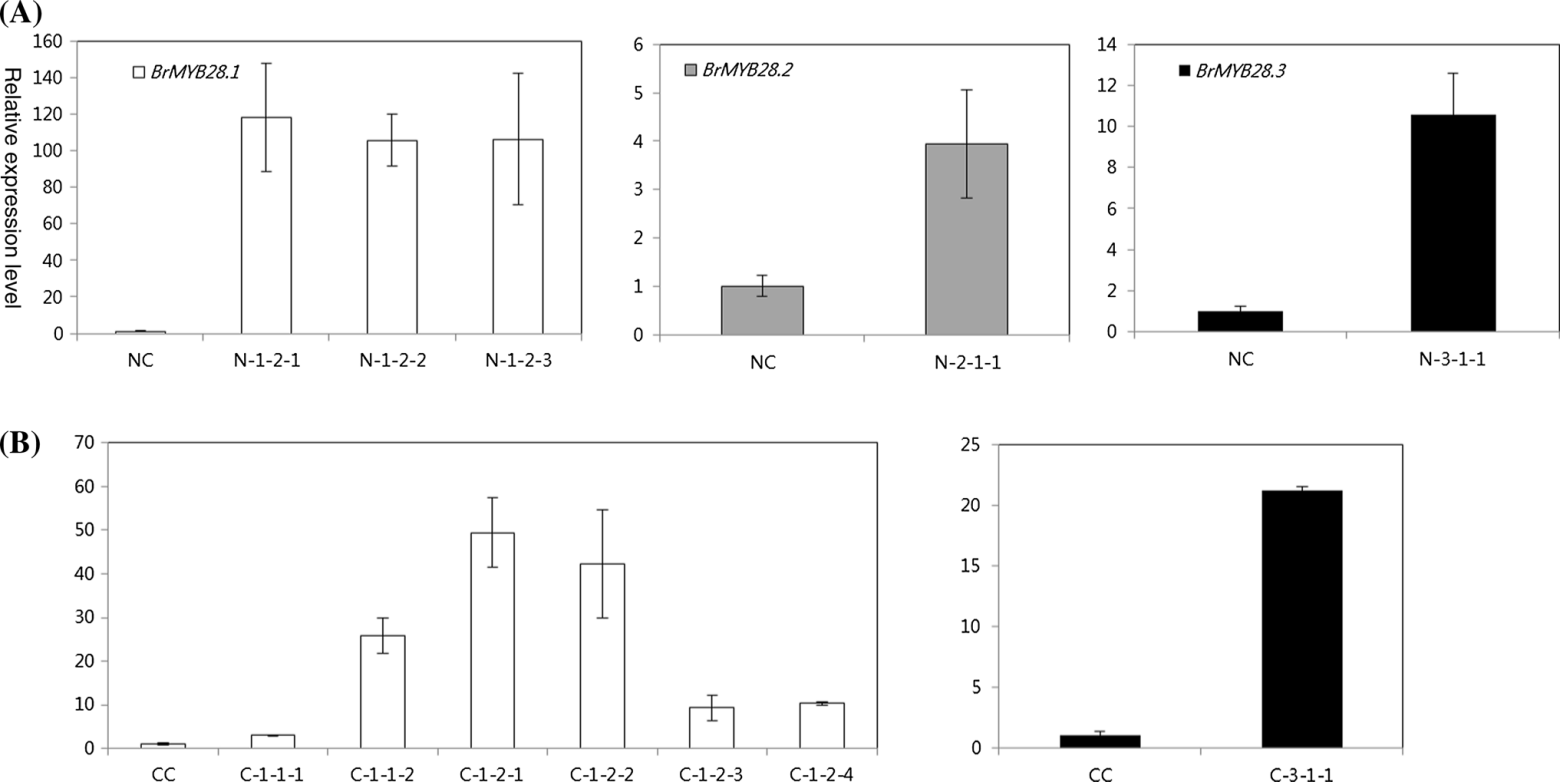
Expression of genes involved in glucosinolate biosynthesis in the 6-week-old leaves of T_2_ transgenic NW (**A**) and CT001 (**B**) plants. Relative expression was determined in triplicate measurements in three independent biological replicates. The *Bractin* gene was used as a quantitative control. NC, CC, non-transgenic plants; N-1-2-1–N-1-2-3, NW *BrMYB28.1* gene transgenic plants; C-1-1-1–C-1-2-4, CT001 *BrMYB28.1* gene transgenic plants; N-2-1-1, NW *BrMYB28.2* gene transgenic plant; N-3-1-1, NW *BrMYB28.3* gene transgenic plant; C-3-1-1, CT001 *BrMYB28.3* gene transgenic plants

**Fig. 8:**
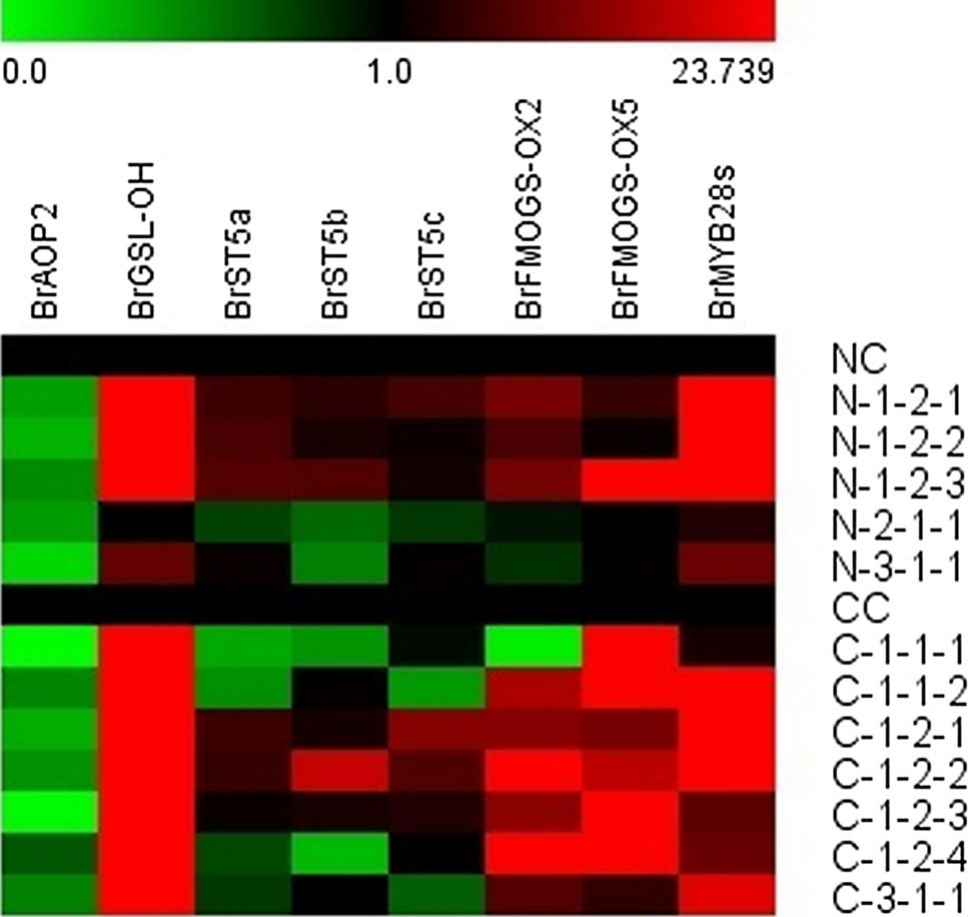
Expression profiling for glucosinolate biosynthesis-related genes in T_2_ homozygous transgenic lines. NC, CC, non-transgenic plants; NC, CC, non-transgenic plants; N-1-2-1–N-1-2-3, NW *BrMYB28.1* gene transgenic plants; C-1-1-1–C-1-2-4, CT001 *BrMYB28.1* gene transgenic plants; N-2-1-1, NW *BrMYB28.2* gene transgenic plant; N-3-1-1, NW *BrMYB28.3* gene transgenic plant; C-3-1-1, CT001 *BrMYB28.3* gene transgenic plants

To assess the different expression of genes for GSL biosynthesis, the expression profiles of genes were investigated in 6-week-old leaves of homozygous T_2_ lines obtained from T_1_ transgenic plants with a segregation ratio of 3:1 by real-time PCR. As shown in Fig. 7, the *BrMYB28.1*, *BrMYB28.2,* and *BrMYB28.3* genes were all significantly up-regulated in the leaves of homozygous transgenic plants. The T_2_ lines showed a significant increase in transcript levels of *BrMYB28* genes, resulting in a significant change in the expression level of GSL biosynthetic genes (Fig. 8, Supplementary Table S5). The expression of *BrGSL*-*OH* genes was significantly increased in transgenic plants with high transcript levels of the *BrMYB28* genes. Expression of the *BrAOP2* gene was significantly decreased compared to that in nontransgenic plants. The *BrMYB28.1*-overexpressing transgenic T_2_ lines N1-2-1, N1-2-2, and N-1-2-3 all showed up-regulation of six GSL biosynthetic genes, except the *BrAOP2* gene. Furthermore, the *BrFMO*_*GS*-*OX2*_ and *BrFMO*_*GS*-*OX5*_ genes were up-regulated in all *BrMYB28.1*-overexpressing CT001 transgenic plants, except C-1-1-1. These results apparently indicate that all three *BrMYB28* genes can function as negative regulators of the *BrAOP2* gene and as positive regulators of the *BrGSL*-*OH* gene.

### Accumulation of GSLs in homozygous T_2_ lines

Homozygous T_2_ transgenic plants with up-regulated expression of all three *BrMYB28* genes showed significant increases in the accumulation of GSLs in leaves. As shown in Table 2, the total GSL content increased in all transgenic plants with overexpression of the three *BrMYB28* genes. All transgenic plants showed markedly increased contents of both aliphatic and indolic GSLs compared to nontransgenic plants. The highest accumulation of total GSLs was observed in two inbred lines with *BrMYB28.1* overexpression: 8.9 µmol/g in N-1-2-3 and 46.8 µmol/g in C-1-2-3. Total GSLs in N-1-2-3 and C-1-2-3 were significantly increased by approximately 4.7- and 4.2-fold, respectively, compared with nontransgenic plants. The total GSL content of N-2-1-1 with *BrMYB28.2* overexpression showed a small increase compared with *BrMYB28.1* and *BrMYB28.3* overexpression. The content of major aliphatic and indolic GSLs was found to be increased in transgenic plants. In contrast, glucoraphanin and sinigrin were only found in the *BrMYB28.1* or *BrMYB28.3* -overexpressing transgenic lines. Furthermore, these homozygous transgenic T_2_ plants showed significantly increased levels of all GSLs compared with T_1_ transgenic plants. Thus, analysis of GSL content and gene expression in transgenic plants suggested that all three *BrMYB28* genes participate in the regulation of aliphatic, indolic, and aromatic GSL biosynthesis.

**Table 2:** GSLs content (μmol g^−1^ dw) in the transgenic T_2_ plants

Trivial name	NC	N-l-2-1	N-l-2-2	N-l-2-3	N-2-1-1	N-3-1-1
Progoitrin	0 ± 0	0.059 ± 0.00	0.050 ± 0.036	0.053 ± 0.043	0.160 ± 0.014	0.147 ± 0.034
Glucoraphanin	0 ± 0	0 ± 0	0 ± 0	0 ± 0	0 ± 0	0 ± 0
Sinigrin	0 ± 0	0.070 ± 0.02	0.047 ± 0.034	0.053 ± 0.038	0 ± 0	0 ± 0
Glucoalyssin	0 ± 0	0.10 ± 0.00	0.074 ± 0.016	0.091 ± 0.013	0 ± 0	0.036 ± 0.051
Gluconapoleiferin	0 ± 0	0.047 ± 0.00	0.036 ± 0.005	0.044 ± 0.006	0 ± 0	0.084 ± 0.080
Gluconapin	0 ± 0	0.08 ± 0.02	0.087 ± 0.012	0.100 ± 0.028	0 ± 0	0.041 ± 0.031
Glucocochlearin	0 ± 0	0.422 ± 0.37	0 ± 0	0.671 ± 0.444	0.151 ± 0.008	0.084 ± 0.060
Glucobrassicanapin	0.034 ± 0.024	0 ± 0	0 ± 0	0 ± 0	0.116 ± 0.084	0.058 ± 0.082
Aliphatic GSLs	0.034 ± 0.024	0.786 ± 0.37	0.295 ± 0.084	1.011 ± 0.460	0.427 ± 0.084	0.450 ± 0.275
Glucobrassicin	1.194 ± 0.274	2.88 ± 0.50	2.954 ± 0.176	3.299 ± 0.194	3.223 ± 0.331	4.119 ± 1.846
4-Methoxyglucobrassicin	0.488 ± 0.117	2.708 ± 0.43	3.435 ± 0.211	3.199 ± 0.531	0.505 ± 0.246	1.480 ± 0.346
Neoglucobrassicin	0.335 ± 0.068	1.10 ± 0.09	0.875 ± 0.062	1.295 ± 0.183	1.102 ± 0.197	0.921 ± 0.272
Indolic GSLs	2.017 ± 0.430	6.694 ± 0.93	7.264 ± 0.430	7.793 ± 0.723	4.829 ± 0.435	6.519 ± 2.401
Gluconasturtiin	0.074 ± 0.003	0.07 ± 0.01	0.124 ± 0.026	0.073 ± 0.018	0.043 ± 0.061	0.239 ± 0.102
Total GSLs	2.124 ± 0.433	7.555 ± 1.23	7.683 ± 0.454	8.876 ± 1.027	5.300 ± 0.365	7.208 ± 2.695

Trivial name	CC	C-l-1-1	C-l-1-2	C-l-2-1	C-l-2-2	C-l-2-3	C-l-2-4	C-3-1-1
Progoitrin	0.094 ± 0.03	2.743 ± 0.075	2.40 ± 0.78	1.16 ± 0.26	3.524 ± 0.727	2.844 ± 0.410	2.509 ± 0.27	1.259 ± 0.65
Glucoraphanin	0 ± 0	0 ± 0	0.045 ± 0.003	0.03 ± 0.02	0.065 ± 0.018	0.049 ± 0.003	0.042 ± 0.00	0.015 ± 0.02
Sinigrin	0.017 ± 0.02	0.13 ± 0.03	0.146 ± 0.024	0.13 ± 0.03	0.140 ± 0.014	0.184 ± 0.037	0.138 ± 0.01	0.098 ± 0.02
Glucoalyssin	0.087 ± 0.03	0.15 ± 0.03	0.163 ± 0.047	0.14 ± 0.04	0.130 ± 0.028	0.229 ± 0.011	0.176 ± 0.01	0.096 ± 0.01
Gluconapoleiferin	0.025 ± 0.03	0.32 ± 0.10	0.696 ± 0.078	0.59 ± 0.13	1.119 ± 0.363	0.719 ± 0.132	0.747 ± 0.09	0.595 ± 0.20
Gluconapin	1.126 ± 0.20	6.65 ± 2.48	8.383 ± 2.992	6.74 ± 2.96	5.988 ± 0.166	10.328 ± 2.091	5.764 ± 0.58	2.637 ± 0.89
Glucocochlearin	0 ± 0	0.09 ± 0.01	0.067 ± 0.009	0.07 ± 0.01	0.062 ± 0.016	0.066 ± 0.011	0.077 ± 0.01	0.113 ± 0.09
Glucobrassicanapin	2.519 ± 0.29	12.55 ± 3.29	17.572 ± 4.936	14.88 ± 5.79	16.488 ± 0.840	21.846 ± 3.047	14.924 ± 1.19	14.715 ± 4.12
Aliphatic GSLs	3.868 ± 0.21	21.04 ± 5.85	29.815 ± 7.774	24.98 ± 9.67	27.516 ± 1.150	36.265 ± 5.321	24.376 ± 2.08	19.528 ± 5.88
Glucobrassicin	4.774 ± 0.87	5.93 ± 0.57	3.368 ± 0.895	3.87 ± 1.29	5.103 ± 2.090	4.904 ± 1.588	6.342 ± 0.78	2.304 ± 0.52
4-Methoxyglucobrassicin	1.448 ± 0.17	2.76 ± 0.07	2.513 ± 0.386	2.81 ± 0.24	2.743 ± 0.344	3.305 ± 0.476	3.178 ± 0.22	2.718 ± 0.70
Neoglucobrassicin	0.764 ± 0.34	1.50 ± 0.16	2.931 ± 0.575	1.38 ± 0.21	1.572 ± 0.819	1.387 ± 0.278	1.948 ± 0.61	6.784 ± 2.08
Indolic GSLs	6.986 ± 1.11	10.20 ± 0.74	8.813 ± 0.645	8.06 ± 1.38	9.419 ± 3.088	9.595 ± 1.815	11.468 ± 1.02	11.806 ± 1.56
Gluconasturtiin	0.296 ± 0.16	0.93 ± 0.06	0.678 ± 0.216	0.52 ± 0.03	0.811 ± 0.256	0.971 ± 0.181	0.574 ± 0.17	0.671 ± 0.31
Total GSLs	11.151 ± 1.30	32.17 ± 5.56	39.305 ± 8.165	33.56 ± 9.67	37.746 ± 4.365	46.831 ± 3.713	36.419 ± 2.99	32.005 ± 5.17

Each value is mean ± standard error (n = 3)

## Discussion

The whole-genome sequencing of *B. rapa* was recently reported (Wang et al. 2011). The results of a comparative analysis with *Arabidopsis* suggest that the triplicated genes of *B. rapa* result from genome triplication via genome evolution following polyploidy. MYB transcription factors are a large gene family of transcription factors in plants and their N termini contain a highly conserved MYB domain (Dias et al. 2003). In particular, the R2R3-MYB transcription factors are known as regulatory proteins in the secondary metabolism of plants (Boddu et al. 2006; Du et al. 2009). R2R3-MYB transcription factors related to the regulation of GSL biosynthesis were recently reported in *Arabidopsis* (Celenza et al. 2005; Gigolashvil et al. 2007). Three members of MYB28, which is known to be a transcription factor that participates in the regulation of aliphatic GSL biosynthesis, were identified in *B. rapa*. The three *BrMYB28* members share 84–85 % nucleotide sequence identity with *AtMYB28* and are located on different chromosomal loci of BAC clones. These results indicate that MYB28 was triplicated by Brassica genome triplication. In the Brassica family, three members of the MYB28 transcription factors are found in *B. rapa*, two in *B. oleracea,* and four in *B. juncea* (Augustine et al. 2013). All *MYB28* genes of the Brassica family also share high levels of sequence conservation with the *AtMYB28* gene of *A. thaliana* and close evolutionary relationships.

The duplication of genes by polyploidy in plants has led to functional diversity as pseudogenes or the gain of additional or novel functions (Adams 2007). In this study, we investigated the expression of three *BrMYB28* genes in different organization and developmental stages of Chinese cabbage by RT-PCR and microarray. *BrMYB28.1* and *BrMYB28.2* showed high levels of expression in seedlings or leaves, with expression patterns differing according to developmental stages. In contrast, *BrMYB28.3* showed no expression in any of the investigated organization and developmental stages of *B. rapa*. Although *BrMYB28.3* showed a high level of sequence homology with *Arabidopsis*, it might be a pseudogene or be subject to epigenetic silencing by randomly occurring polyploidy in the genome. The results of protein alignment analysis shown in Fig. 1 suggested the possibility that insertion of a repetitive sequence of ‘F’ amino acid in the C-terminal region might lead to the pseudogenization of the *BrMYB28.3* gene. These results also support recent reports that gene duplication in *B. rapa* and *Arabidopsis* may allow functional diversification during evolution by changing protein structures (Du et al. 2012). However, it is uncertain whether *BrMYB28.3* is a pseudogene resulting from the insertion of repetitive sequences with no expression in any of the organizational or developmental stages of *B. rapa*. In addition, the differential and various expression patterns between *BrMYB28.1* and *BrMYB28.2* suggest that internal sequence divergence of paralogs may result in the functional change of duplicated genes.

We observed changes in the function of the three *BrMYB28* transcription factors by polyploidization of the *B. rapa* genome. The *BrMYB28* genes were identified to increase in expression levels during GSL accumulation in all of T_1_ and T_2_ lines of transgenic Chinese cabbage. Although transgenic plants contained the same construct, the expression levels of three *BrMYB28* genes differed markedly in independent T_1_ and T_2_ transgenic plants. Consequently, the differences in the increase of expression level of *BrMYB28* genes induced high GSL accumulation compared to nontransgenic plants. Additionally, there was no similar correlation between the expression of *BrMYB28* genes and glucosinolate content. Such a lack of correlations between mRNA levels and levels of the final target product has also been shown in other studies (Emani et al. 2003; Takahashi et al. 2010). Some studies have demonstrated that variation in expression levels are caused by difference in transgene integration sites (Matzke and Matzke 1998; Kohli et al. 2006). Thus, we can speculate that the different expression levels of the *BrMYB28* genes are attributable to differences in the transgene integration site.

We also found that the total GSL content in homozygous T_2_ lines of transgenic CT001 was significantly increased compared to the heterozygous parental transgenic lines, whereas the total GSL content of T_2_ lines of transgenic NW were similar to their heterozygous parental transgenic lines. In particular, five homozygous T_2_ lines overexpressing *BrMYB28.1* exhibited a high content of total GSLs and expression of glucoraphanin. Duan et al. (1996) reported that the homozygous transgenic plants produced a higher content of foreign protein compared to heterozygous transgenic plants in some transgenic plants. The similar GSL content in T_1_ and T_2_ of NW transgenic lines indicates that the variation in GSL content by the overexpression of *BrMYB28* genes can differ depending on the genotype in *B. rapa*. Overexpression of *BrMYB28* genes regulated the mRNA levels of *BrAOP2* and *BrGSL*-*OH* in transgenic T_2_ plants. Although MYB28 and MYB29 have been reported as positive regulators of aliphatic GSL biosynthetic genes in *Arabidopsis* (Sønderby et al. 2010), in the present study, all three *BrMYB28* genes were shown to function as negative regulators of the *BrAOP2* gene. This suggests that regulation of GSL biosynthesis by MYB28 may differ depending on the plant. Furthermore, accurate observation of changes in the expression levels of more paralog genes will be required to determine the expression network of the GSL biosynthesis genes regulated by the three *BrMYB28* transcription factors. Analysis of the transgenic plants showed that *BrMYB28.3* is fully functional for GSL biosynthesis, similar to *BrMYB28.1* and *BrMYB28.2*. Zou et al. (2009) found evidence of expression for a few pseudogenes, although with lower expression levels compare to functional genes in Arabidopsis and rice. Our findings indicate that *BrMYB28.3* has a unique role as an expressed pseudogene that regulates GSL biosynthesis.

Gene silencing of the *BnAOP2* gene has been reported to cause a reduction in progoitrin and an increase in glucoraphanin in the seeds of *B. napus* (Liu et al. 2012). Furthermore, the GSL-OH gene was shown to be involved in the accumulation of progoitrin in *Arabidopsis* (Hansen et al. 2008). Therefore, in the present study, the increased level of progoitrin in all T_2_ transgenic plants and only glucoraphanin in overexpressed CT001 transgenic plants caused by overexpression of *BrMYB28.1* and *BrMYB28.3* may be due to the decreased expression level of the *BrAOP2* gene and the increased expression level of the *BrGSL*-*OH* gene. We can consider various possibilities, such as total GSL content, different genotype, interaction with paralog genes of *BrAOP2* and *BrGSL*-*OH*, or other functions of *BrMYB28s,* as reasons for the detection of glucoraphanin only in the *BrMYB28* genes-overexpressing lines of CT001 T_2_ transgenic plants.

The three BrMYB28 proteins are involved in regulating the biosynthesis of all aliphatic, indolic, and aromatic GSLs in transgenic plants of two inbred lines of *B. rapa*, although there are sequence differences among the paralogs. The overexpressing transgenic plants clearly showed that all of the three *BrMYB28* participate in controlling the accumulation of both short- and long-chain GSLs. Recent studies on GSL biosynthesis-related genes have reported that MYB28 is involved only in the aliphatic GSL biosynthesis pathway (*AtMYB28* of *A. thaliana* and two *BjMYB28* genes of *B. juncea*). This finding indicates that the two *BjMYB28* genes resulting from polyploidization of the *B. juncea* genome showed no function other than regulation of aliphatic GSL biosynthesis in transgenic *A. thaliana*. Therefore, the different accumulation patterns of GSL biosynthesis between *B. rapa* and *A. thaliana* suggests that some different mechanisms may operate in the GSL biosynthesis pathway of these plant species. The constant functionalization of *BjMYB28* paralog genes may also be related to the functional analysis of GSL biosynthesis using transgenic *Arabidopsis*.

We have successfully developed Chinese cabbage containing high levels of GSL by overexpressing *BrMYB28* genes in *B. rapa,* which is recalcitrant to *Agrobacterium*-mediated transformation. Our results clearly indicate that all of the three *BrMYB28* genes are related to GSL biosynthetic processes in *B. rapa*. The high GSL contents of homozygous T_2_ plants promoted by increased expression of *BrMYB28* genes will prove useful for studying the regulation of complex GSL biosynthesis pathways in polyploid plants and in subsequent functional studies that will examine the anti-carcinogenic activity and defense properties of GSLs.

## Electronic supplementary material

Below is the link to the electronic supplementary material.

Supplementary material 1 (XLSX 27 kb)

Supplementary material 2 (PPTX 133 kb)
