# Supplementary material for: Functional analysis of three BrMYB28 transcription factors controlling the biosynthesis of glucosinolates in Brassica rapa
Source: Plant Mol Biol. 2016 Jan 28;90(4):503–16. doi: 10.1007/s11103-016-0437-z (PMC4766241; doi:10.1007/s11103-016-0437-z)
Supplement: Supplementary file 2 — Supplementary material 2 (PPTX 133 kb) [file 11103_2016_437_MOESM2_ESM.pptx]

## Slide 1
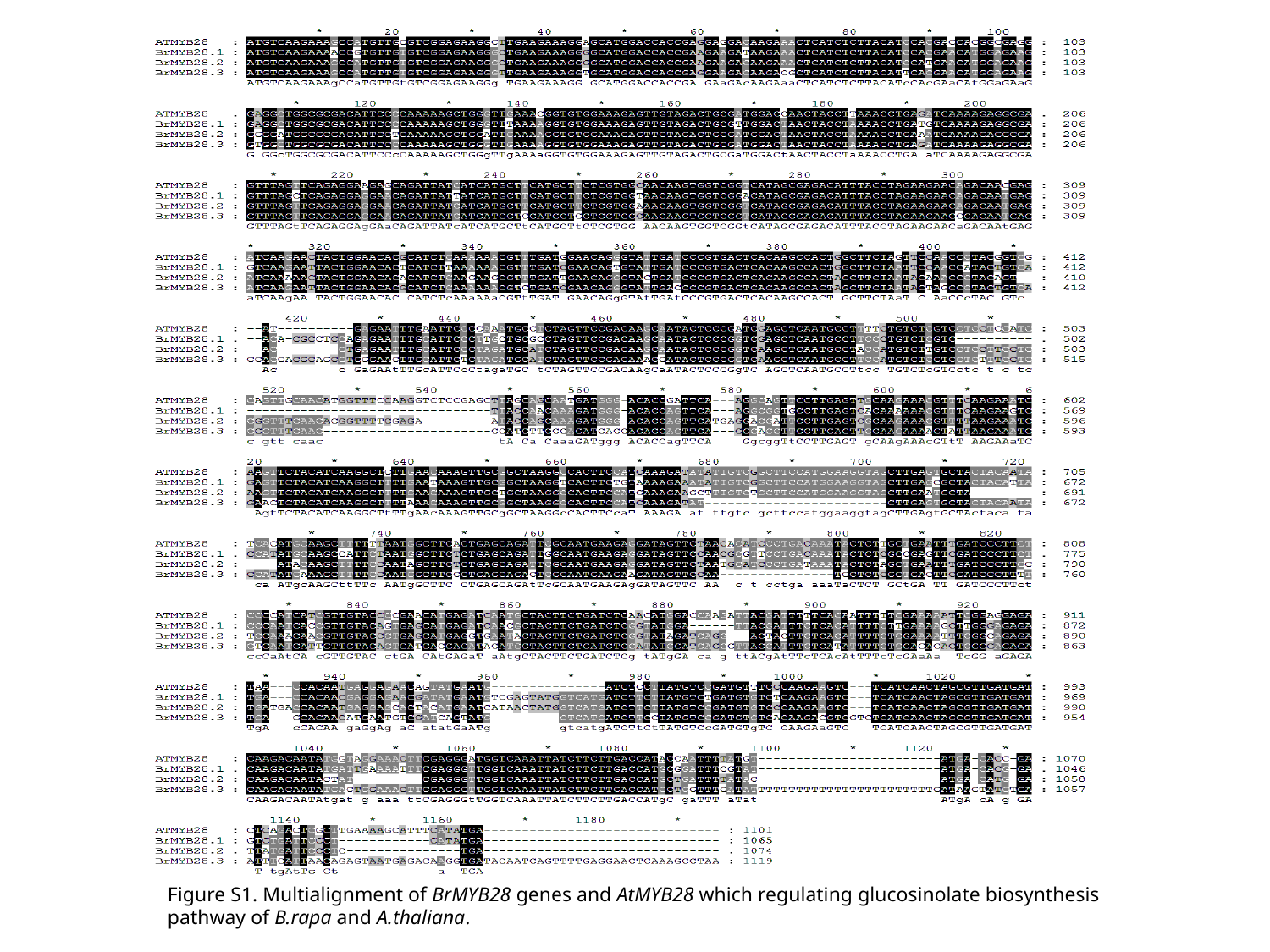

Figure S1. Multialignment of BrMYB28 genes and AtMYB28 which regulating glucosinolate biosynthesis pathway of B.rapa and A.thaliana.
